# The Short-Term Dynamics of Peers and Delinquent Behavior: An Analysis of Bi-weekly Changes Within a High School Student Network

**DOI:** 10.1007/s10940-017-9340-2

**Published:** 2017-03-14

**Authors:** Frank M. Weerman, Pamela Wilcox, Christopher J. Sullivan

**Affiliations:** 1 NSCR (Netherlands Institute for the Study of Crime and Law Enforcement), P.O. Box 71304, 1008 BH Amsterdam, The Netherlands; 2 School of Criminal Justice, University of Cincinnati, Cincinnati, OH USA; 3 Criminology Department, Erasmus School of Law, Erasmus University, Rotterdam, The Netherlands

**Keywords:** Delinquency, Peers, Social networks, Short term dynamics, Adolescence

## Abstract

**Objectives:**

To analyze short-term changes in peer affiliations, offending behavior and routine activities in order to evaluate three different processes: peer selection, peer socialization and situational peer influences.

**Methods:**

The short-term longitudinal TEENS study was conducted among a cohort of students from one mid-sized high school in Kentucky, as part of the larger Rural Substance Abuse and Violence Project. The study sample consists of one complete network of 155 ninth graders who completed surveys about their peer affiliations, routine activities and offending behaviors over the course of five waves of data collection during the beginning of the school year. The measurement intervals were no more than 2 weeks long. Longitudinal network analysis (SIENA software that enables actor-oriented stochastic modeling) was used to estimate peer selection, socialization, and situational effects.

**Results:**

Peer networks, offending, and routine activities appeared to be very volatile over the research period. Peer selection effects were found for structural network properties, demographics and delinquent values, but not for peer delinquency. We did not find significant peer socialization effects within the research period, but instead found that changes in offending were related to situational changes in unstructured socializing, alcohol use and marijuana use.

**Conclusions:**

The results suggest that traditional time lags of one year or six months between measurements may fail to capture short-term relations between peers and behavior. Long-term peer influence processes like socialization may be less important in the short run, while situational peer effects might be more salient.

## Introduction

Adolescence is a dynamic period, in which individuals mature physically, psychologically, and socially (e.g., Coleman and Hendry 1999; Steinberg 1996). It is also a period in which the salience of peer influence increases and the time that is spent with peers peaks (Berndt 1979; Warr 1993; Csikszentmihalyi and Larson 1984), and it is a period in which involvement in delinquent behavior increases and peaks as well (Farrington 1986; Hirschi and Gottfredson 1983; Sweeten et al. 2013). The dynamic nature of adolescence also reveals itself in the relative instability of peer relations. Adolescent friendships are often not long lasting, but rather volatile, with substantial changes in peer networks across or within school years (Berndt and Hoyle 1985; Cairns and Cairns 1994; Değirmencioğlu et al. 1998), and even within the course of months (Chan and Poulin 2007). As Chan and Poulin (2007, p. 579) state, “Youths’ social universe represents a system that is constantly in motion in which friendship relations are formed, sustained, or split up on a regular basis.”

There are also reasons to believe that delinquent behavior during adolescence is highly dynamic. After all, delinquent behavior is, by nature, not continuous: even the most delinquent adolescents will abide the law most of the time. Exemplifying this, data from space–time budget interviews show that active adolescent offenders commit crimes in only 2% of the hours that they are awake (Bernasco et al. 2013; see also Wikström et al. 2010, 2012). And data from research employing life history calendars suggest that periods of high delinquent activity can be alternated with periods of low or no activity (Horney et al. 1995; Slocum et al. 2005; Uggen and Thompson 2003).

Despite our knowledge of the short-term volatility of both peer interactions and delinquency, studies linking these two types of fluctuations are limited. Many studies have reported a relation between delinquent behavior of friends and self-reported delinquency of adolescents (see e.g. Haynie 2001; Matsueda and Anderson 1998; Warr 2002), or a relation between time spent with peers in unstructured and unsupervised activities (referred to as “unstructured socializing”) and self-reported delinquency (Osgood et al. 1996; Osgood and Anderson 2004; Weerman et al. 2013). However, the large majority of these studies have either relied on cross-sectional data or on longitudinal data with relatively long time intervals. Therefore, we do not know how short-term changes in peer relations and peer-related activities are related to short-term changes in offending.

More generally, major longitudinal studies in criminology (see Thornberry and Krohn 2003) have followed up young people over time with time lags of usually one year, or at best six months. Studies that collect social network data on peers and delinquency behavior have typically employed time intervals of one year (e.g. Baerveldt et al. 2008; Haynie and Osgood 2005; Weerman 2011), or at best three months (Knecht et al. 2010). One study has investigated monthly changes in (ego) peer networks of adolescents (Chan and Poulin 2007), but this study did not include data on delinquent behavior.

Time lags of several months to one year may be well suited to capture long term developments during adolescence. However, they do not grasp the immediate short-term changes that seem to characterize peer relationships and behavior in adolescence. Hence, linking peer relations to behavior with the usual time lags may lead to mis-estimations since the peer network characterizing one point in a school year is likely quite detached from behavior measured several months or a year later. More generally, processes underlying the relationship between peers and behavior may happen at a much quicker pace than implicitly assumed in existing studies. Friendship selection and adaptation of one’s own behavior to that of peers may take place within a timeframe of weeks or even days, instead of many months or years. Some peer processes are even assumed to take place immediately, without any delay (see e.g., Short 1998; Warr 2002).

In short, what is missing from the literature about peers and delinquency are studies that capture short-term (e.g., monthly or weekly) changes—what Warr (2002) called the “micro life course”—in describing the various ways in which peers might influence delinquent behavior. The current study is a first attempt to explore such short-term changes and what they reveal about three different peer processes: peer selection, peer socialization, and situational peer influences. We analyze short-term changes in peer affiliations, routine activities such as unstructured socializing and substance use, and offending behavior within a single school network of 9th graders from Kentucky. Change is assessed over the course of five waves of data collection during the beginning of the school year. In sum, we employ much shorter time intervals between waves than previous studies on peers and delinquency: instead of several years or months, our study covers a time period of no more than 10 weeks, with measurements of peer networks, behavior and time use over the course of five waves, with 2-week intervals between each of them.

## Theoretical Background: Peer Selection, Peer Socialization and Situational Mechanisms

Although empirical findings about the association between peers and delinquency are undisputed (see e.g., Warr 2002), the process of identifying their underlying mechanisms^1^ has led to strong theoretical debate. For a long time, the debate centered on the causal ordering between exposure to delinquent peers and committing delinquent acts. In criminology, control theorists argued that the association between delinquent peers and delinquency was spurious and, for the most part, the result of selection of delinquent friends by youths that were already delinquents (Hirschi 1969; Gottfredson and Hirschi 1990). It was suggested that “birds of a feather flock together” (Glueck and Glueck 1950). On the other hand, social learning theorists, like Akers (1973, 2009), argued that the robust relationship between peers and delinquency was evidence for a peer influence effect (see also Warr 2002). This debate reflects a more general awareness in the social sciences that similarity between peers in behavior, attitudes, and taste can be the result of *peer selection* based on similarity (or “homophily”) as well as *peer influence* (see e.g., McPherson et al. 2001; Steglich et al. 2010; Veenstra and Dijkstra 2011). Nowadays, most scholars take the stance that both peer selection and peer influence processes are relevant (e.g. Thornberry 1987; Smith and Ecob 2013; Warr 2002).

Within the peer influence perspective, scholars have also debated the mechanisms by which peers affect each other toward delinquency. A classical point of view is that peers adopt each other’s norms and attitudes about behavior (i.e., they are a source of normative influence). Sutherland (1947) argued that “definitions” (cognitions, attitudes, techniques) that leave room for deviant behavior can be learned in intimate groups and will be transmitted when there is an excess of such definitions over definitions that forbid deviant behavior. Building on general learning principles, Akers (1973) suggested that peer influences also take place through other ways of “social learning”: peers can provide social reinforcement (i.e., approval, acceptance) for acts of crime, or peers who have been rewarded for delinquency can serve as models for imitation. Recently, McGloin (2009) built on balance theory (Heider 1958) and suggested that peers may exert influence because adolescents seek delinquency balance with their close friends.

The three previous mechanisms of peer influence have in common that they do not happen instantaneously. Rather, they all need some time to come into play—at least several days or weeks. Adolescents need to interact with each other to transmit their attitudes and definitions about delinquency, or to reinforce or sanction each other’s behavior, and adolescents need time to observe each other’s behavior and adapt their own behavior in order to achieve balance. We refer to these time-consuming mechanisms of peer influence, collectively, as *peer socialization mechanisms*.

There are also peer influence mechanisms that take place immediately, in the spur of the moment, without need for personal development. We refer to these immediate mechanisms as *situational peer mechanisms*. Osgood et al. (1996), building on the routine activities approach in criminology (Cohen and Felson 1979), suggested that “unstructured socializing” with peers in the absence of adults increases delinquency by offering immediate opportunities and rewards for offending. The company of peers may offer symbolic rewards of status and reputation, and peers may serve as co-offenders or provide the means for an offense. The absence of adults means that there are no capable guardians around to prevent delinquent acts from happening. Warr (2002) described several processes that may stimulate delinquent behavior in groups of young people immediately and magnify the delinquent behavior of one to all. In particular, fear of ridicule from peers, the wish to show loyalty to friends, and status competition among peers are strong conformity mechanisms that may explain delinquency, without a need for adolescents to adopt norms condoning it. Additionally, Warr stresses the importance of collective processes taking place in peer groups, such as anonymity, diffusion of responsibility, and rowdiness.

Another situational mechanism that may be at play in groups of adolescents is the disinhibiting effect of alcohol and drug use. The use of alcohol and other drugs may lead to a temporary lowering of self-control and, as a consequence, to antisocial or imprudent behavior. Several studies show that alcohol intake is linked directly to delinquent behavior (see Fals-Stewart 2003; Felson et al. 2008); though it has been noted that the effects of marijuana are less clear (Fals-Stewart et al. 2003; Myerscough and Taylor 1985). Although substance use is not a peer influence in itself, the intake of alcohol and drugs among adolescents occurs in the company of peers in most cases (Erickson and Jensen 1977; Warr 1996). Warr (2002, p. 80) states that “alcohol and marijuana are used by adolescents almost exclusively in group settings.” Further, it has been suggested that situational group mechanisms and collective processes may be facilitated by the presence of alcohol and drugs (Miller 2013). Thus, in this study, we view alcohol and marijuana use as potential situational peer mechanisms, along with unstructured socializing.

## Network Dynamics and Peer Selection

In many studies on the relation between peers and delinquency, the concept of peer selection has been narrowly studied, focusing on peers’ similarity in delinquent behavior only (e.g. Kandel 1978; Matsueda and Anderson 1998), or peers’ similarity in the direct causes of delinquent behavior, like low self-control (Creemers et al. 2010; Gottfredson and Hirschi 1990). However, the process of selecting and changing friendships is much broader than what is addressed in such studies; several additional mechanisms must be taken into account when studying the short-term dynamics of peer networks.

First, there are general structural mechanisms or “laws” that appear to happen in most social networks. A classic example (e.g. Simmel 1910) is the tendency for *network closure.* In general, when an individual (Person A) has a link to a friend (Person B), this individual (Person A) tends to become friends with others (Persons C) who are already friends with his or her friend (Person B). Likewise, when an individual (Person A) has chosen someone as a friend (Person B), the friends (Persons C) of this person tend to choose the individual (Person A) as a friend over time. This can be explained by a person’s preference for *cognitive balance* in their relationships with others (Heider 1958), but it may also be simply an effect of an enhanced opportunity to become acquainted with “friends of a friend.” Another example of a structural network mechanism is the tendency to *reciprocate* friendship choices of other persons. If somebody nominates a person as a friend at a certain moment in time, it is highly likely that this nomination will be reciprocated over time by the person who is chosen.

Secondly, there are also other similarity preferences that shape the making and breaking of friendships among peers. One of the most important preferences is the tendency of adolescents to become friends with others from their own sex, which is referred to as gender homophily in the literature (Benenson et al. 1998; McPherson et al. 2001). Adolescents may also have a tendency to select others from the same ethnic background as themselves, or with similar attitudes and with similar abilities as themselves (see e.g., Liska 1978; Reed and Rose 1998). In fact, all kinds of personal attributes and characteristics have been found to act as criteria that may lead to homophily in friendship selection (see Veenstra and Dijkstra 2011).

Lastly, certain characteristics may lead to a higher level of popularity or activity in friendship selection. Some adolescents are clearly more interesting than others, and are thus more often liked or nominated as a friend by their peers. For example, adolescents who report a number of anti-social behaviors appear to be more popular among their classmates, and at the same time, they nominate fewer others as friends (Dijkstra et al. 2009). Alcohol consumption also has been found to make adolescents more popular among their peers (Kreager et al. 2011; Osgood et al. 2013), as have the characteristics of physical attractiveness and athleticism (Dijkstra et al. 2009). Likewise, there are also adolescents who are more active in selecting friends and liking or nominating others as friends. For example, girls have been found to be more active in social networks than boys (e.g., Weerman and Bijleveld 2007). Further, people in general seem to be attracted to those who are already popular. This tendency, which has been referred to as the “Matthew effect,” suggests that those who already have nominations as friend shall be given more. In one study, this effect was reported among young children, where it was found that during the school year, children tended to choose more often other children that were relatively popular (Schaefer et al. 2010).

## Previous Network Studies on Peers, Activities, and Delinquent Behavior

Specialized research designs and analytical methods have led to greater insights regarding the dynamics of peer networks and delinquent behavior. In particular, the increase in longitudinal studies that collect data on complete networks of adolescents (e.g., school classes/cohorts) and the use of dynamic stochastic network models have been major developments. For example, Simulation Investigation for Empirical Network Analyses—or SIENA modeling, developed by Snijders and colleagues (Ripley et al. 2014; Snijders 1996; Snijders et al. 2010)—has allowed for substantial analytic refinement. Using such tools, much has been learned about the dynamic nature of peer networks, the extent to which peers select each other based on delinquency or other preferences, and, alternatively, the extent to which adolescents adapt their behavior over time to that of their peers in the network.

Studies using these sophisticated network analytic designs are consistent regarding several aspects of the process of adolescent peer selection. For example, a number of studies report a preference for similarity in gender and ethnicity (Haynie et al. 2014; Veenstra and Dijkstra 2011; Weerman 2011). Additionally, structural network effects on peer selection are usually reported in this body of research, including reciprocity, transitivity, and balance (see Ripley et al. 2014; Haynie et al. 2014). Also, a negative “three-cycles-effect” has been found which may be interpreted as a preference for hierarchy in the friendship nominations in which Person C does not choose Person A when Person A chooses Person B and Person B chooses Person C (Ripley et al. 2014; Snijders et al. 2010).

Findings about the role of delinquency in the selection of friends are less consistent among studies using network analytical methods. Some studies report significant and clear selection effects of delinquent behavior, while others do not. For example, Knecht et al.’s (2010) analysis of students from school classrooms found that similarity in delinquency affected selection of peers. Similarly, Snijders and Baerveldt (2003) found effects of similarity in delinquency on tie formation as well as effects of dissimilarity in delinquency on the breaking of ties. Haynie et al. (2014), employing data from the Add Health Study, also found clear selection effects for violent as well as non-violent delinquency. In contrast, Burk et al. (2007) and Baerveldt et al. (2008) reported only small selection effects for similarity in delinquency. Analyzing the NSCR school project data, Weerman (2011) did not find similarity in delinquency to affect selection of peers at all, after controlling for a wide range of other similarity preferences and structural network effects.

Prior social network studies are also mixed regarding the extent of the *influence* of peer delinquency, net of selection and other network effects. Several studies report no influence effect—in other words, peer delinquency did not affect own delinquency (e.g., Knecht et al. 2010). Others found modest but significant effects (e.g., Baerveldt et al. 2008; Weerman 2011) and some (e.g., Burk et al. 2007; Haynie et al. 2014) found significant influence effects that were relatively strong.

The mixed results regarding selection and influence described above emerged from social network studies that differ in important respects methodologically. A few studies included several waves of data spanning relatively short periods of time. For example, the study by Knecht et al. (2010) included three waves of data during one school year, collected with intervals of three months. Other studies used two waves, with a 1-year interval separating data-collection points (e.g. Weerman 2011; Haynie et al. 2014). Some studies collected network data from school classes (Knecht et al. 2010), while others used data from complete school grades (Weerman 2011; Haynie et al. 2014) or data from a snowball sample of adolescents from a few school classes and their nominated peers (Burk et al. 2007). These differences in design, data collection, and analytic approach may be partly responsible for the variation in outcomes across studies (see also Veenstra and Dijkstra 2011).

## The Current Study

As illustrated above, while adolescent peer relations and behavior patterns are characterized by considerable short-term volatility, most longitudinal studies on the relation between peers and delinquency have relied on data with relatively long time intervals between successive waves. These studies may uncover long-term developmental patterns during this period but cannot capture the short-term dynamics that may be just as important in understanding the mechanisms underlying the often observed relationship between peers and delinquency. Indeed, research employing social network data is hampered by the use of time intervals ranging from several months to 1 year. Social network data that are collected with shorter time intervals than usually employed may shed new light on the dynamics of peers and delinquency. Further, short-term data on peer-oriented activities may offer insight on the role of situational peer influence processes, which are seldom investigated alongside processes of peer selection and influence.

Thus, the contribution of the current study is that it uses short-term social network data, based on five waves of bi-weekly data collection, to study changes in peer relations, activities, substance use, and delinquency. It uses such short term longitudinal data to analyze and distinguish three different processes: peer selection, peer influence due to peer socialization processes, and peer influence due to situational peer processes. More specifically, we examine the following research questions:

1. How much short-term change takes place within each investigated period of 2 weeks with regard to (a) peer relations within an overall school grade network; (b) the amount of unstructured socializing; (c) the use of alcohol and marijuana; and (d) involvement in different types of offending (minor and serious violence, property/vandalism)?
2. Which potential friendship preferences and structural network effects contribute to changes in the network among peers (i.e., selection) over the short research period? What is the role of peer delinquency in these changes?
3. Consistent with the idea of peer influence via peer socialization, are respondents adapting their involvement in delinquent behavior to the average level of their friends’ delinquency?
4. Consistent with the notion of peer influence via situational mechanisms, are changes in delinquent behavior related to changes in unstructured socializing and alcohol/marijuana use over the short research period?

We take the stance that peer selection processes, peer socialization mechanisms, as well as situational peer effects, may occur simultaneously during the 2-week data-collection intervals used in our study. In line with the volatile and dynamic character of adolescence that has been reported previously, we expect to see substantial change in the peer networks, activity patterns, substance use and offending behaviors during each wave. With regard to selection, the evidence of prior research is mixed; delinquent behavior may play a role in the changes in friendship nominations that will occur in our research period, but alongside other mechanisms of peer selection. With regard to peer socialization mechanisms, the literature on longitudinal network analysis suggests that these influences may be traced more easily with several waves of network data with relatively short time intervals (Ripley et al. 2014), so we expect these effects to occur in our sample. Finally, we expect that delinquent behavior will fluctuate immediately with the time that students spend engaged in unstructured socializing and with the use of alcohol and marijuana in each 2 week period.

## Methods

The data that are used in the current paper come from a short-term longitudinal study of the 9th-grade cohort of students from one mid-sized high school in Kentucky. The site of the study was a school that was originally part of the Rural Substance Abuse and Violence Project—a large, 4-year study of adolescents in schools across Kentucky that consisted of annual surveys of a sample of nearly 4000 students within 60 schools at baseline (NIDA grant DA-11317, Richard Clayton, PI). As a follow up to that project, investigators purposively selected one school in a small county from their original, larger sample of Kentucky schools in order to study short-term change in peer networks. This was the only high school in the county, and there is only one middle school in the county that feeds students to the selected high school. This means that most students enrolled in the sampled high school had previously attended middle-school together as well.

This single-school follow-up study is referred to as the TEENS project (TEENS serving as an acronym for Teenage Networks in Schools). In the TEENS project, self-report baseline surveys were administered by project staff in the homeroom period during the second week of classes in the 2006–2007 academic year. All students in the school from whom active parental consent was obtained participated in the baseline survey.^2^ Subsequent to this baseline survey of all students, five follow-up surveys were administered, in 2-week increments, to the 9th grade cohort only. For the purposes of this study, we focus only on the 9th-grade cohort which was studied longitudinally.

The 9th-grade cohort consisted of 213 students in total. Active parental consent was obtained from 84% of these students, yielding a cohort sample size of 178.^3^ At baseline, respondents in this 9th-grade sample ranged from 14 to 16 years of age (mean = 14.37, sd = 0.56). Among the 178 students from whom active parental consent was obtained, respondent assent and completion rates ranged from 88 to 95% over the course of the six observation periods (baseline, plus five follow-ups). The analysis presented here is based upon the 155 students within the 9th grade cohort who participated in the first five waves of data collection. We chose to disregard data from the sixth wave, because this wave had a relatively high dropout rate (only 133 respondents participated in this wave). The data from the five waves that are included allow for a unique exploration of the potentially changing character of peer relations within almost the entire network of ninth-grade students. While sample generalizability is limited, it is noteworthy that the county in which the sampled high school is located is representative, demographically speaking, of the counties in the Commonwealth of Kentucky.^4^

In addition to asking about friendships, the group-administered survey contained questions about demographic and background characteristics (i.e., self-control; delinquent values), self-reported delinquency, unstructured socializing, and substance use. Together, these measures allowed for the examination of stability versus change in the school’s social network, peer selection processes, as well processes of peer influence, including both peer socialization and situational effects.

## Measures

### Social Network Measurement

Each survey (baseline and 2-week follow up surveys) asked students to provide the names of their *five closest friends*, regardless of whether the friends attended the school. Because the high school sampled for TEENS was the only public high school within the county, it provided a context in which most nominated friends would be students at the same school, thus allowing researchers to capture a large amount of data about friends from the friends themselves.

On average, the respondents from the current study sample (155 ninth graders who participated in at least in the first and the fifth wave) named almost four “closest friends” within their school (mean = 3.96; sd. = 1.40).^5^ However, only the respondents from the 9th grade of the school completed the bi-weekly follow-up surveys. Therefore, we restricted our measurement of the respondents’ social network to nominated friends within the 9th-grade cohort of the high school that was selected for the longitudinal portion of the study. This restriction means that we are missing part of social world of the adolescents, namely the friends they have in higher grades and outside of school. On average, the students nominated 1.12 friends that were not from the ninth grade and who could thus not be included in our network analysis directly (although we included a control variable for the number of friends respondents nominated in higher grades). Restricting ourselves to the 9th grade enabled us to capture a *complete* network with clear boundaries (the school’s ninth grade student network), which, in turn, allows for the use of dynamic stochastic network models. In our sample, respondents nominated on average about three respondents from their own 9th school grade that were also included in the sample (mean = 2.84, sd = 1.50).^6^ This is sufficient to conduct a SIENA analysis to model network changes and peer processes.

### Offending

At each bi-weekly measurement point, students were queried about their offending during the previous 2 weeks. More specifically, students were asked to identify the number of days over the previous 2 weeks that they had participated in 10 different delinquent activities. Response categories for each behavior ranged from 1 = “0 days” to 7 = “13 or more days”.^7^ These ten survey items were used to construct three dichotomous measures of different offending types and one total scale of offending type variety. This way of scaling was chosen to arrive at dependent variables with limited ranges and limited heterogeneity, both of which are advised for SIENA modeling (see Ripley et al. 2014).^8^ The three different types of offending that we distinguished were: minor violence, serious violence, and property offending/vandalism.^9^

*Minor violence* (1 = yes; 0 = no) referred to participation in behaviors including pushing/grabbing/shoving someone, punching/hitting/slapping someone, or threatening to hit or hurt someone. Table 1, which displays descriptive statistics for study variables, shows that the prevalence rate of minor violence varied across waves, ranging from 21% at wave 5 to 38% at wave 1. *Serious violence* (1 = yes; 0 = no) measured whether or not the respondent had intentionally injured someone to the point that medical attention was required, had carried a gun or other weapon, or had used a gun or other weapon to threaten or injure someone. Rates of participation in serious violence were also variable across waves, ranging from 3% (wave 3) to 9% (waves 2 and 5) of students reporting such participation. Finally, *property offending/vandalism* ((1 = yes; 0 = no) measured whether the respondent had stolen money or property worth less than $50, stolen money or property worth more than $50, stolen a car or motor vehicle, or had vandalized public or private property.^10^ Descriptive statistics indicate that property crime prevalence ranged from a low of 4% (wave 3) to a high of 11% (wave 2).

**Table 1:** Descriptive statistics

Variable (min–max)	Wave 1		Wave 2		Wave 3		Wave 4		Wave 5	
	Mean	SD	Mean	SD	Mean	SD	Mean	SD	Mean	SD
Stable covariates										
Sex (1 = female)	0.50									
Race (1 = non white)	0.17									
SES (1–7)	3.89	1.52								
School attachment (1–5)	3.54	0.63								
Delinquent values (1–4)	1.43	0.72								
Low self control (1–4)	1.87	0.71								
Previous offending	0.23	0.83								
Changing covariates										
Unstructured socializing (5–30)	11.19	4.61	10.81	4.80	10.50	4.69	10.18	4.62	10.57	5.39
Alcohol use (1 = yes)	0.27		0.16		0.12		0.09		0.09	
Marijuana use (1 = yes)	0.09		0.08		0.05		0.06		0.07	
Friends from higher grades	0.88	0.94	0.61	0.88	0.97	1.10	1.01	1.09	0.99	1.04
Dependent variables										
Count of offending categories (0–3)	0.51	0.74	0.52	0.82	0.38	0.60	0.38	0.73	0.37	0.78
Minor violence (1 = yes)	0.38		0.32		0.31		0.26		0.21	
Serious violence (1 = yes)	0.05		0.09		0.03		0.04		0.09	
Property crime/vandalism (1 = yes)	0.09		0.11		0.04		0.08		0.07	

Based on these three offense categories, we also constructed a measure of *offending variety* by summing the number of offending categories (ranging from zero to three) in which respondents had participated during each 2-week increment between waves. Descriptive statistics presented in Table 1 indicate that mean offending variety ranged between 0.37 and 0.52, with the highest means in the first two periods of the study (which measured behavior occurring during summer break and behavior occurring during the first 2 weeks of the school year).

### Unstructured Socializing

In order to measure *unstructured socializing* at each wave, we summed five survey items asking respondents how many hours during the previous 2 weeks they had:(1) spent time with friends; (2) gone to parties without an adult around; (3) gone to the movies; (4) gone to the mall; and (5) drove or rode around with friends. These activities are closely in line with Osgood et al.’s original formulation of the concept of unstructured socializing. In fact, each of these items was among the thirteen activities that were included in the original measurement of unstructured socializing (Osgood et al. 1996). Response categories for each of the summed items ranged from 1 = “none” to 6 = “50 + hours,” resulting in a summed index with potential values between 5 and 30. Descriptive statistics provided in Table 1 indicate that mean levels of overall unstructured socializing ranged between 10.18 (at wave 4) and 11.19 (at wave 1, referring to the summer break). These figures suggest that sampled respondents, on average, spent roughly 1–10 hours every 2 weeks in unstructured socializing.

### Substance Use

In addition to the measure of unstructured socializing, we also included two measures of substance use that often takes place within the company of peers: 1) a dichotomous measure of *alcohol use* (1 = yes; 0 = no) over the course of the previous 2 weeks, and 2) a dichotomous measure of *marijuana use* (1 = yes; 0 = no) during the previous 2 weeks.^11^ Table 1 shows that prevalence of alcohol use varied substantially across waves, ranging from 9% (waves 4 and 5) to 27% (wave 1, which refers to the summer break). Prevalence of marijuana use was less variable, yet still ranged from 5% (wave 3) to 9% (wave 1).

### Control Variables

The measurements of friendship networks, offending, unstructured socializing, and substance use are the primary input for the SIENA analysis and will be used to analyze peer selection, peer socialization, and situational processes (analysis strategy described in more detail below). Beyond these key variables, we also included assessments of respondents’ sex, race, socioeconomic status, school attachment, delinquent values, and low self-control. Above and beyond similarity in offending behavior, similarity in respondent versus peers’ sex, race, SES, school attachment, delinquent values, and low self-control may affect peer nominations. Likewise, it is possible that these variables also affect the amount of change in delinquent behavior among the respondents in addition to the change potentially caused by peer delinquency and situational variables. Therefore, it is important to include them as “control” variables.

We also included a variable for involvement in offending during the previous school year. It is possible that respondents base their selection of friends not only on current delinquent behavior of their peers but also (or merely) on previous delinquent behavior of peers. It is also possible that previous involvement in offending has an effect on the probability that respondents increase their involvement in offending during the 10 week study period. Finally, we also control for the number of respondents’ friends who are in higher grades (e.g., 10th, 11th, or 12th grades). We calculated this control variable for each wave of the study (as a time-varying covariate) and estimated its potential effect on (changes in) involvement in offending, under the assumption that having older friends might increase delinquent opportunities and group influences. We also included the number of friends in higher grades as a potential effect on friendship selection from one’s own grade. Below we provide detailed information about each of the control variables.

*Sex* and *race* were measured as dichotomous variables (1 = female, 0 = male; 1 = non white, 0 = white). Table 1 indicates that the sample was evenly divided between males and females, whereas the majority was white as opposed to non-white (83 vs. 17%, respectively). *Socioeconomic status* (*SES*) was measured at baseline, or wave 1, as the average level of education of respondents’ parents, with scores ranging from 1 (grade school or less) to 7 (graduate of professional school). The sample mean for SES fell roughly in the middle of the range of possible scores, at 3.89 (sd = 1.52).

*Attachment to school* is measured at wave 1 by averaging responses to six survey items asking respondents how strongly they disagreed or agreed, on a response scale ranging from 1 to 5, with statements about the strength of their relationships with teachers, the importance of education, and their attitudes toward school. Higher scores represent stronger attachment, and the overall sample mean of 3.54 (sd = 0.63) suggests that students were, on average, moderately attached to school. *Delinquent values* was measured at wave 1 as the mean score on 12 items asking respondents how strongly they felt about the wrongness of certain behaviors, including cheating on school tests, damaging property, stealing property, hitting someone, using drugs, and selling drugs. Scores range from 1 (“very wrong”) to 4 (“not wrong at all”). Descriptive statistics presented in Table 1 indicate that students tended to see these behaviors, on average, as falling somewhere between “very wrong” and “somewhat wrong” (mean = 1.43, sd = 0.72). *Low self*-*control* was measured at wave 1 as the average score across 11 survey items asking students to report, on a scale ranging from 1 to 4, the extent to which they experienced problems with temper control, restlessness, attention, frustration, and nervousness. The sample mean score of 1.87 (sd = 0.71) indicates that students, on average, fell near the midpoint of the measurement range.

*Previous offending* was measured in wave 1 of the study, as the average score across 10 survey items asking respondents to report their level of involvement in different offending types during the previous school year, with response for each item ranging from 1 (0 days) to 7 (100 or more days). The types of offenses are the same as those used to construct the offending outcomes of minor violence, serious violence, and property crime/vandalism. *Friends from higher grades* was measured as the number of students from higher grade that were nominated as a friend by the respondent. Since the ninth grade is the first grade of this high school, all nominated friends from the same high school outside one’s own grade are necessarily from higher grades. Because friendships nominations could change during the study period, we constructed this control variable for each wave separately. On average, respondents had 0.89 friends from higher grades in each wave (sd = 1.01).^12^

## Analytic Strategy

Though the descriptive statistics presented in Table 1, and summarized above, provide some indication of change in several key time-varying covariates, the initial step in our analysis was to describe the pattern of change across waves in greater detail. First, we visually illustrate the changes with regard to the peer nominations by presenting a graphical representation of the actual friendship ties comprising the network of participating 9th graders at (two) different waves (employing NetDraw software, see Borgatti 2002). Next, we provide a tabular summary of the changes across the waves with regard to friendship choices, offending types, unstructured socializing, and substance use. For each of these key variables, we present the levels of stability (i.e., same friendship nominations or stability at “zero” or stability at previous rate) and patterns of change (i.e., changing friendship nominations and increasing/decreasing levels or stopping/starting certain behaviors) across waves.

After the descriptive analysis of the short-term changes in peer relations, behavior, and activities, we turn to our explanatory analysis of peer selection processes, peer socialization processes, and situational processes. To enable simultaneous estimation of these processes, we used RSiena (SIENA run in R) version 4.0 (Ripley et al. 2014). The SIENA (Simulation Investigation for Empirical Network Analysis) method enables estimation of effects on individual changes in network ties as well as effects on changes in behavior. Instead of relying on respondent perceptions about peer behavior, it uses direct information of the nominated peers in the network themselves. It includes estimations of various effects on peer nominations and behavior, including not only peer delinquency but also structural network effects, peer selection based on personal characteristics other than offending, and effects on behavior of stable and dynamic covariates other than peer delinquency.

In the modeling procedure, the total observed change (in friendship ties and behavior) between the first and the last measurement moment is modeled into small basic changes, so-called micro-steps. A network micro-step entails the breaking or making of one tie with another person (i.e., a peer nomination); a behavioral micro-step is a one unit change in the dependent variable of interest (in the current paper, offending). The changes over time are modeled as a Markov process in which sequential stages are dependent on the previous situation. The sequences of these micro-steps are used to estimate the parameters in a network simulation process (using a Markov Chain Monte Carlo approach). Network dynamics and behavioral changes are modeled simultaneously by taking the estimated state of the changing network as a constraint for the behavioral changes and vice versa (for more details see Snijders et al. 2010; Ripley et al. 2014). Parameter sizes and standard errors are estimated by comparing the simulations with the observations within each wave.^13^

In the network dynamic part of the SIENA modeling procedure, we included several potential *effects on peer selection.* First, we estimated a number of structural network effects that are often reported to contribute to network evolution (see Veenstra and Dijkstra 2011; Ripley et al. 2014). This included the basic outdegree effect (the baseline probability of making ties with others); the effects of reciprocity, transitive ties, and balance; the 3-cycles effect; and the indegree popularity effect (also referred to as the Matthew effect). Compared to previous applications these are relatively many structural network effects, but exploratory analyses suggested that they might all be relevant and that combining them would not result in convergence problems.^14^

Further, we estimated several selection effects based on peer characteristics, including selection effects based on similarity in sex, race, SES, school attachment, delinquent values, low self-control, and previous and current offending. These effects indicate to which extent fellow school students are more often nominated as new friends and/or more often retained as friends when they have characteristics that are similar or the same as the respondent. Most of these selection effects were based on peer characteristics that were measured only at wave 1. However, the selection effect of similarity in current peer delinquency is estimated in a dynamic way, and simultaneous with the influence effect of peer delinquency. The dynamic effect of peer delinquency represents the classic peer selection effect referred to in the criminological literature as “birds of a feather flock together” (e.g. Hirschi 1969): it indicates whether peers who are similar in delinquent behavior at a particular moment have a higher chance to become (or stay) friends.

We also included so-called ego and alter effects for each of the aforementioned peer characteristics. Ego-effects indicate whether a respondent with a particular characteristic is more active in nominating peers (from the ninth grade); alter-effects indicate whether others with a particular characteristic more often receive nominations (i.e., whether they are more popular as a friend).

In the behavioral part of the model, *effects on changes in offending* are estimated. First, we included two basic SIENA parameters representing the general trend in offending behavior over the five waves of the study: the “linear shape,” which is a baseline estimation of the average tendency over the research period, and the “quadratic shape,” which indicates the effect of initial offending behavior on itself.^15^

Second, we included parameters to control for effects of several personal characteristics that were measured in the initial wave, namely sex, race, socioeconomic status, school attachment, delinquent values, low self-control, and previous offending. These effects indicate the extent to which these personal characteristics are related to the chance that a respondent increases or decreases his or her involvement in offending during the study period. We also included a parameter in the model to control for an effect of having older friends in school on the chance to increase or decrease involvement in offending. This refers to the effect of the number of older friends in a particular wave (it is modeled in SIENA as a changing covariate).

Third, we included *socialization and situational effects* in the model. Peer socialization was assessed by the ‘average similarity’ effect in the SIENA model. This parameter estimates the extent to which respondents adjust their offending towards the average level of offending of their nominated peers in the network (i.e. the extent to which they become more similar to the average of their alters).^16^ This parameter is calculated by modeling and simulating the changes in behavior over the complete study period into micro-steps that are needed to get from the situation in the first wave to the situation in the last wave, which simultaneously takes into account the simulated micro-changes in the network composition. This means that the ‘average similarity’ effects are not only based on the observed values of peer delinquency at wave one, but also on the simulated levels in the periods between waves, which are compared to the actual observations of each wave to arrive at parameter estimates and standard errors.

Situational peer processes were assessed by modeling the contemporaneous effects of levels of unstructured socializing and substance use (alcohol as well as marijuana use) on offending. These variables were measured at each of the five waves and included as changing covariates in the RSiena procedure. To ensure that we really modeled the contemporaneous relations, we did not estimate lagged effects of changing covariates on behavior, as is the standard in RSiena, but instead we estimated effects of the situational variables on offending within the same 2 weeks. Unlike the socialization effects, the estimation of the situational effects is not modeled simultaneously with network and behavior changes in RSiena, but based on the youths’ reported levels of unstructured socializing and substance use at each wave.^17^

The model that is outlined above was estimated first with the total offending variety measure as the outcome. Then we re-estimated the model, substituting total offending with each of the three measures reflecting separate types of offending as dependent variables. Finally, we conducted a robustness check on the outcomes of the SIENA analysis to scrutinize whether the different reference period of the first wave of the study (the summer break instead of the previous 2 weeks) had biased the results. This test consisted of a re-run of all RSiena analyses with exclusion of the data from the first wave (that referred to the summer break). The sample for the re-run contained data from 150 persons who participated in the second as well as the fifth wave of the TEENS study.

## Results

### Changes in Peers and Behavior

**Fig. 1:**
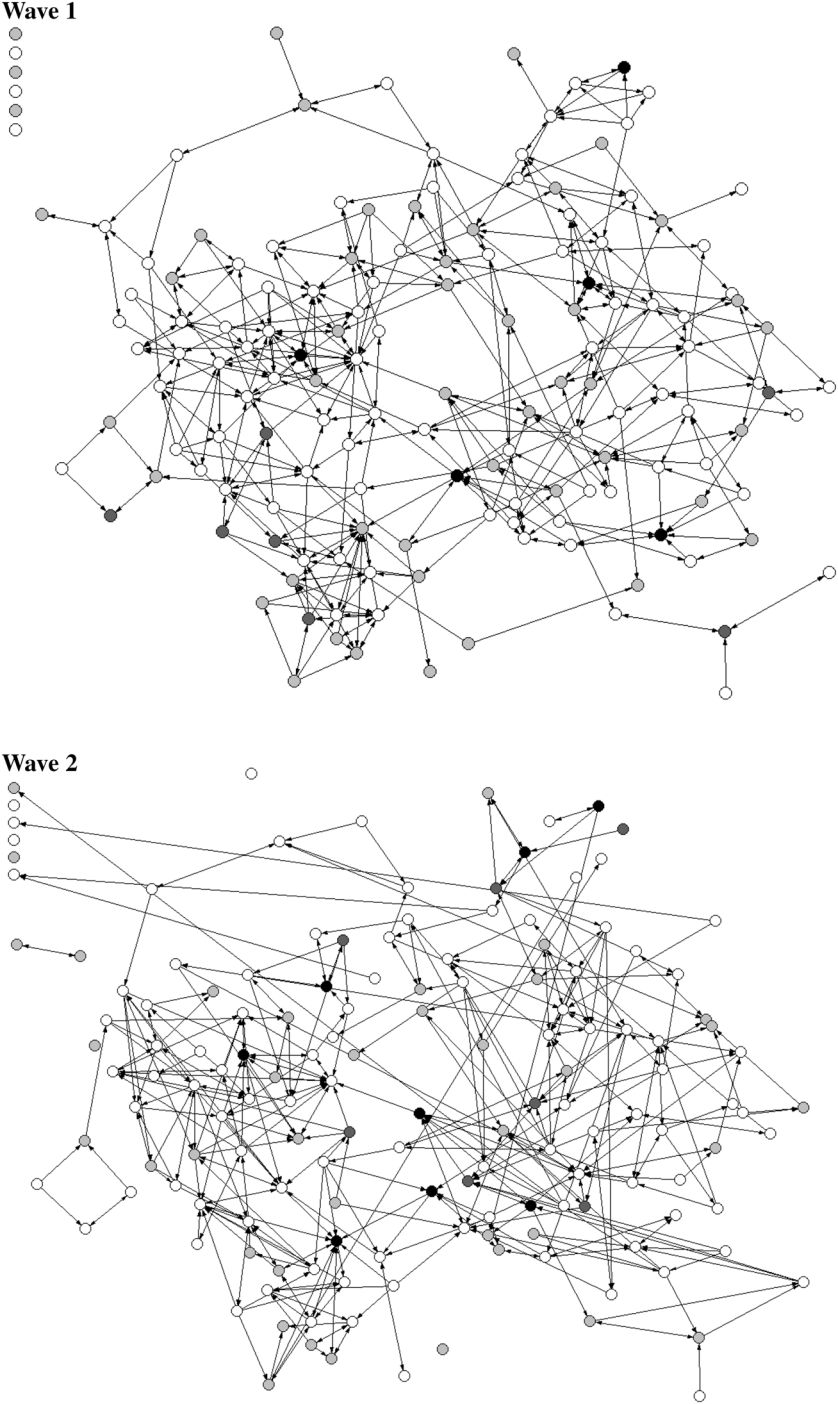
Ties and behavior of the 9th grade network at Wave 1 and Wave 2. *White circles* represent non-offenders, *grey circles* represent respondents who reported one type of offending, *black circles* represent respondents who reported two or three types of offending

The first step in our analysis is to investigate how much change actually takes place during the relatively short period of our data collection. Figure 1 offers a first impression of the changes in peer relations within the 9th grade while also depicting the offending levels of the students in the network. The figure displays the ties among the sample of 9th grade students at waves 1 and 2. In the figure, the circles represent the students and the arrows represent the friendship nominations (one-directional indicating non-reciprocated ties, or two-directional, representing reciprocated ties). Similar figures were created for the other waves of data collection, and these show a similar pattern.

Figure 1 offers a visual sense of the highly dynamic nature of the network. While a portion of the ties within the network remain intact between waves 1 and 2, many other ties break during this timeframe, and many others form anew. Taken as a whole, the network established at wave 1 seems to be scrambled by wave 2. Thus, even within the short time period of 2 weeks, friendship ties are clearly subject to change. Figure 1 also shows that offending behavior is due to change within this short period of time: although some of the circles depicted in the networks have the same shading in wave 2 compared to wave 1, many others change from white to grey or black, or vice versa.

Table 2 presents descriptive data that gives a more detailed sense of the changes occurring within the five bi-weekly waves of the study, not only with regard to ties but also to offending as well as situational influence measures. The values in Table 2 represent the frequency distributions across categories of stability and change for each 2-week increment.

**Table 2:** Prevalence of stability versus change in ties, offending, and situational variables between each wave

	Wave 1 → 2		Wave 2 → 3		Wave 3 → 4		Wave 4 → 5	
	%	n	%	n	%	n	%	n
Friendship ties								
Stable ties	43.1	(248)	47.8	(251)	51.1	(279)	52.2	(288)
New ties	25.5	(147)	26.9	(141)	24.4	(133)	23.9	(132)
Broken ties	31.4	(181)	25.3	(133)	24.5	(134)	23.9	(132)
Offending								
Total variety								
Stable not involved	48.5	(65)	56.6	(73)	61.2	(82)	66.2	(92)
Stable involved at same rate	17.9	(24)	20.2	(26)	17.9	(24)	10.8	(15)
Increased involvement	14.9	(20)	10.9	(14)	7.5	(10)	10.1	(14)
Decreased involvement	18.7	(25)	12.4	(16)	13.4	(18)	12.9	(18)
Minor violence								
Stable not involved	51.1	(70)	60.0	(75)	63.0	(85)	66.2	(92)
Stable involved	21.2	(29)	17.6	(22)	19.3	(26)	13.7	(19)
Increased involvement	11.7	(16)	12.0	(15)	5.2	(7)	7.9	(11)
Decreased involvement	16.1	(22)	10.4	(13)	12.6	(17)	12.2	(17)
Serious violence								
Stable not involved	89.6	(124)	91.7	(121)	95.6	(129)	91.4	(128)
Stable involved	89.6	(124)	2.3	(3)	2.2	(3)	3.6	(5)
Increased involvement	5.8	(8)	1.5	(2)	1.5	(2)	4.3	(6)
Decreased involvement	2.2	(3)	4.5	(6)	0.7	(1)	0.7	(1)
Property/vandalism								
Stable not involved	85.5	(118)	90.8	(119)	93.3	(125)	90.7	(127)
Stable involved	5.1	(7)	2.3	(3)	2.2	(3)	5.0	(7)
Increased involvement	5.8	(8)	1.5	(2)	3.7	(5)	1.4	(2)
Decreased involvement	3.6	(5)	5.3	(7)	0.7	(1)	2.9	(4)
Situational variables								
Alcohol use in period								
Stable non-use	68.8	(97)	82.1	(110)	84.7	(116)	86.7	(124)
Stable use	11.3	(16)	9.0	(12)	5.8	(8)	4.2	(6)
Increase	5.0	(7)	3.7	(5)	3.6	(5)	4.2	(6)
Decrease	14.9	(21)	5.2	(7)	5.8	(8)	4.9	(7)
Marijuana use in period								
Stable non-use	87.1	(122)	92.5	(124)	92.7	(127)	90.2	(129)
Stable use	5.0	(7)	5.2	(7)	3.6	(5)	4.2	(6)
Increase	2.9	(4)	0.0	(0)	2.9	(4)	2.9	(5)
Decrease	5.0	(7)	2.2	(3)	0.7	(1)	2.1	(3)
Unstructured socializing								
Stable	37.2	(51)	35.2	(45)	40.5	(53)	40.6	(56)
Increase	27.0	(37)	27.3	(35)	31.3	(41)	29.7	(41)
Decrease	35.8	(49)	37.5	(48)	28.2	(37)	29.7	(41)

The frequencies displayed in Table 2 show that stability in non-ties is clearly the norm. However, a substantial number of new ties form while old ties break across each of the increments separating the five waves of data collection. In fact, taken together, the number of changing ties (new and broken ties) is even higher than the number of stable ties between each wave, supporting the impression of a highly dynamic network.^18^ The network seems to get a little more settled towards the end of the period (i.e., the number of stable ties increases), but even then, changing ties are still more abundant than stable ties in this short period of time.

Table 2 also shows the frequency distributions across categories of stability and change in offending for the sampled cases. For all types of offending, stable non-involvement in offending is the norm, while stable involvement characterizes a minority of students. There is also a considerable amount of change in offending. Between each wave of data collection, a substantial minority of students became newly-involved in offending—in comparison to no involvement at the previous wave, their involvement increased. Other students who had been involved in offending did not offend in the next wave; thus, their involvement decreased. Such change is especially evident for minor violence and the overall measure for offending variety. Between each wave, a substantial number of respondents changed involvement in these offense types (between 18 and 29% of the sample in each wave). Even for the less common behaviors of property/vandalism and serious violence, a substantial number of respondents increased or decreased involvement between waves of data collection.

The last section of Table 2 presents descriptive data on patterns of stability versus change in the situational variables. The patterns for alcohol and marijuana use are similar to those observed for offending. Most cases exhibit stability in non-use within the 2-week increments comprising the entire study period, yet there is also evidence of both increase and decrease in use among some students. Unstructured socializing appears to be the most volatile of the situational variables, with more change than stability in each of the 2-week increments covered in the study.

### Network Analysis of Peer Selection, Socialization, and Situational Influences on Overall Offending

Table 3 shows the results from our SIENA analysis of network change (peer selection) and behavioral change (peer and other influences). The table presents coefficient estimates and standard errors for effects on peer selection (making and breaking ties) and for effects on the overall offending (variety) measure (increasing or decreasing involvement).

**Table 3:** SIENA estimation of network and behavior dynamics of total offending variety: parameter estimates and standard errors (n persons = 155; n observations = 755)

Parameter	Estimate	SE
Network structural effects		
Outdegree/density effect	−3.377**	(0.117)
Reciprocity	1.933**	(0.107)
Transitive ties	0.931**	(0.080)
Balance	0.116**	(0.019)
3-cycles effect	−0.183^+^	(0.103)
Indegree popularity	0.057**	(0.014)
Other preferences/selection effects		
Alter female	0.046	(0.073)
Ego female	0.157^+^	(0.090)
Same sex	0.611**	(0.067)
Alter minority	−0.033	(0.103)
Ego minority	−0.099	(0.107)
Same race	0.115	(0.097)
SES alter	0.020	(0.024)
SES ego	0.016	(0.027)
Similarity in SES	0.275	(0.194)
School attachment alter	0.063	(0.058)
School attachment ego	−0.072	(0.070)
Similarity school attachment	0.278	(0.176)
Delinquent values alter	0.162*	(0.068)
Delinquent values ego	0.031	(0.072)
Similarity in delinquent values	0.564**	(0.204)
Low self-control alter	−0.031	(0.055)
Low self-control ego	−0.254**	(0.064)
Similarity in low self-control	−0.055	(0.174)
Number of friends in higher grades alter	0.062	(0.041)
Number of friends in higher grades ego	−0.043	(0.047)
Similarity in # of friends in higher grades	0.513*	(0.220)
Previous offending alter	0.107	(0.078)
Previous offending ego	0.153^+^	(0.083)
Similarity in previous offending	0.719	(0.479)
Behavioral dynamic selection effect		
Offending alter	0.039	(0.098)
Offending ego	0.082	(0.118)
Similarity in offending	−0.177	(0.360)
Effects on offending: basic, controls		
Linear shape (trend)	−1.296**	(0.221)
Quadratic shape	0.010	(0.194)
Sex	−0.050	(0.262)
Race	0.362	(0.290)
SES	0.034	(0.089)
School attachment	−0.347	(0.213)
Delinquent values	0.121	(0.172)
Low self-control	0.139	(0.193)
Number of friends in higher grades	−0.337*	(0.167)
Previous offending	0.062	(0.189)
Effects on offending: dynamic socialization effect		
Average similarity (socialization effect)	−0.565	(1.535)
Effects on offending: situational effects		
Unstructured socializing during period	0.048*	(0.023)
Drinking alcohol during period	0.844*	(0.331)
Using marijuana during period	1.054*	(0.529)

^+^ *p* < .10; * *p* < .05; ** *p* < .01

The first set of parameters refers to basic and structural network effects on peer selection; these coefficients suggest that almost all of the estimated network effects are strong and significant. Next to the basic outdegree effect (not making ties to anyone), there is a very clear tendency to reciprocate friendships, to make transitive ties with students who are indirectly connected to a person, to get balance in nominations (making friends with someone who chooses an existing friend), and to nominate students who are relatively popular (receiving many nominations). The negative 3-cycles effect (indicating preference for hierarchy in the ties) is one-tailed (or marginally) significant (*p* < .10). Although it is difficult to interpret the magnitude of the structural network effects, most of them seem to be very robust, which suggests that they are basic drivers of the short-term network dynamics of our sample.

The second set of effects displayed in Table 3 indicates the effects of respondent and peer characteristics on peer selection (except actual offending behavior, or ‘behavioral selection’, discussed below). It appears that same-sex preferences are substantial and statistically significant: when students make new friends, boys tend to choose boys and girls tend to choose girls. There is a one-tailed significant ego effect of sex, indicating that females are more active in making new friendships than males in the sample. The absence of an alter effect of sex means that boys and girls have equal probabilities to be chosen as a friend by others. There are no significant preference effects for either race or socioeconomic status, and neither did school attachment of respondents and peers affect friendship preferences. However, there are preferences surrounding delinquent values. There is a substantially significant similarity effect as well as a significant alter effect. These effects indicate that respondents prefer to become friends with peers who have values about offending similar to themselves, and that students with delinquent values receive more nominations as friends than others.

The findings further indicate that there is a significant negative ego effect for self-control, indicating that students with relatively low levels of self-control establish fewer new friendships than others. There is also a significant similarity effect for the number of friends from higher grades on peer selection indicating that respondents tend to nominate friends with similar amounts of older friends as themselves.

Finally, there is a one-tailed, significant ego effect of previous offending, which means that respondents that were relatively more involved in offending in the past are more active in making new friendships. However, we did not find a significant alter or similarity effect for previous offending, which suggests that previous offending does not make alters more popular as friends, and also that respondents do not tend to nominate each other on the basis of their past offending behavior.

The third section of Table 3 displays the behavioral selection effects, which reflect the degree to which offending behavior reported by the respondents in one of the five waves of this study affects peer selection at the next. These parameters are modeled simultaneously with peer influences on offending behavior (discussed below). The results indicate that neither of the behavioral selection effects is statistically significant. This implies that offending behavior does not play a substantial role in making or breaking friendships in the 9th grade sample during the research period. In other words, delinquent students do not tend to ‘flock together’ within the timeframe of our study.

The fourth section in Table 3 presents the basic parameters and potential effects of control variables on change in overall offending. The first basic parameter, the linear shape effect, is significant. The negative sign of this parameter indicates that there is a general tendency for overall offending to decrease during the research period (this reflects the decreasing means for offending from Table 1). The quadratic shape effect is not significant, which means that there is no tendency for differences in offending between the students to increase during the research period.

Only one of the control variable effects is statistically significant (or marginally significant). The number of friends in higher grades has a significant negative effect on offending, indicating that respondents with more friends in higher grades are less inclined than others to increase their offending during the 10-week study period.^19^

In contrast, there is no statistically significant effect of sex, race, SES, school attachment, delinquent values and self-control on changes in offending during the five waves of the study. Also offending in the previous school year did not affect the chance of becoming increasingly involved in offending during the 10 week study period.

The fifth section in Table 3 presents the peer socialization and situational effects on overall offending. The first parameter of this section, modeled simultaneously with the behavioral selection effect (discussed above), represents the classic peer socialization influence. This ‘average similarity effect’ is not statistically significant, which means that there was no tendency for respondents to adjust their level of overall offending to the average of their friends.^20^ Thus, we did not find a short-term peer socialization effect during the research period.

The next three parameters represent the situational effects. Interestingly, these all reach significance. Changes in unstructured socializing, drinking, and marijuana use during the 2 week periods are all positively related to changes in offending. The estimate for unstructured socializing indicates an average change in offending variety of about 0.23 for a one standard deviation change in unstructured socializing.^21^ The estimated effects of alcohol and marijuana use (a 0.844 and 1.054 change in offending variety when someone moves from no use to use) are even more substantial.

### Effects for the Three Offense Categories

Table 4 shows the results from re-estimations of the SIENA model reported in Table 3 in which the three different types of offending are outcome variables in place of the total offending variety measure. In these models, the behavioral selection and socialization effects refer to the specific offense categories of minor violence, serious violence and vandalism/property offenses, respectively. Only the selection and influence effects with regard to different offense categories are presented in Table 4. The outcomes for the non-behavioral peer selection effects and the basic and control effects on different types of offending are similar or almost identical to those presented in Table 3.^22^

**Table 4:** SIENA estimation of network and behavior dynamics for three types of offending: parameter estimates and standard errors (n persons = 155; n observations = 755)

Parameter	Minor violence		Serious violence		Vandalism/property offending	
	Estimate	SE	Estimate	SE	Estimate	SE
Behavioral selection effect						
Offending alter	0.036	(0.143)	−0.626	(2.320)	−0.510	(1.327)
Offending ego	0.016	(0.150)	0.043	(2.225)	−0.482	(1.226)
Similarity in offending	−0.212	(0.180)	−0.996	(2.627)	−0.746	(1.370)
Effects on offending: socialization and situational effects						
Average similarity (socialization)	−0.256	(0.746)	−0.313	(2.680)	−1.063	(3.693)
Unstructured socializing period (sit.)	0.093*	(0.039)	0.121**	(0.049)	−0.113	(0.110)
Drinking alcohol during period (sit.)	0.924	(0.595)	0.256	(1.099)	3.485**	(1.188)
Using marijuana during period (sit.)	1.469	(1.008)	2.560*	(1.142)	2.552	(1.692)

^+^ *p* < .10; * *p* < .05; ** *p* < .01

Table 4 indicates that there are no behavioral selection effects for the three types of offending, which is in line with our finding for the overall offending measure. This means that students from our sample did not have a preference to become friends with others who are similar in minor violence, similar in serious violence or similar in vandalism/property offending. The results presented in Table 4 also show that there is no significant average similarity effect for each of the three offense categories. This is in line with our finding for overall offending where we did not find short-term socialization effects either. On the other hand, we did find several situational effects regarding the three offense categories. Interestingly, the categories differ in which situational parameters are significant. For minor violence, there is a significant effect for unstructured socializing, but alcohol and marijuana use exert non-significant effects. For serious violence, there is a significant effect of unstructured socializing and marijuana use, but the effect of alcohol use is absent. For vandalism/property offending, the effect of alcohol use is significant and substantial, whereas effects for unstructured socializing and marijuana use are not. Altogether, unstructured socializing appears to have a stronger relationship with violence, while alcohol use is more strongly associated with non-violent offending. While the findings also suggest that marijuana use seems to be related to serious violence only, it should be noted that the estimated effect sizes for marijuana use across the other offense categories are similar.

### Robustness Check

As a robustness check, we re-estimated all SIENA models excluding the data from the first wave of measurement, which referred to the summer break as the reference period instead of the two previous weeks. The findings, which are presented in Appendices 1 and 2, were quite similar to those presented above, but a few differences emerge.

In the final model for overall offending (Appendix 1), the 3-cycles effect (preference for hierarchy in the ties) and the ego female effect (more activity of girls in making ties), which were already relatively weak in our main findings, became non-significant. The effect of preference for similarity in having friends in higher grades went from significant to marginally significant, while the ego effect (more activity in making ties) of previous offending went from marginally significant to significant. There was also a marginally significant preference effect for having the same race in the re-estimation. With regard to effect on offending, the effect of friends in higher grades became non-significant. A more remarkable difference is that the effect of marijuana use on overall offending is now non-significant, while it was significant in the previous estimation. The effect of unstructured socializing on overall offending became marginally significant (*p* < .10) instead of significant at *p* < .05.

Most of these are minor differences, and the major findings are very stable. We also found robust structural network effects, substantial preferences for similarity in sex and delinquent values, and again no preferences for similarity in offending in the re-estimation for the period of wave 2 to wave 5,. There was no socialization effect of peer offending in those models, but the situational effects were present though less pronounced.

The models for the offenses types separately (Appendix 2), are also very similar to the analyses presented above. We did not find a behavioral selection effect for either of the offense types, nor did we find a socialization effect. We did again find several situational effects, with some slight differences from the offense-specific results presented earlier. We found modest effects of unstructured socializing on minor and serious violence similar to those reported in the main analysis, but with the latter effect significant at *p* < .10 as opposed to *p* < .05. We also found a substantial effect of concurrent alcohol use on vandalism and property offending, as in the main analysis. However, unlike the main analysis, there was an effect of marijuana use on vandalism/property offending, but not on serious violence.

## Discussion

Adolescence is a period that is characterized by swift changes in peer relations, activities and behavior. Yet, the relationship between peers and delinquency has typically been studied as if adolescent development moves slowly, with relatively long-term intervals between measurements. In this article, we studied the development of adolescents of one ninth grade school network during the first 10 weeks of a school year, employing uniquely short time intervals between measurements of no more than 2 weeks. In each wave, data were collected about peer relations, time use, substance use, and offending. This enabled us not only to describe the dynamics of peers and behavior during this focused time window, but also to study three potential peer processes that may explain the relationship between peers and delinquency: selection, socialization, and situational effects.

First of all, the results demonstrate the highly dynamic nature of peer networks during the investigated period of adolescence. The peer relations within the investigated ninth grade peer network sample appeared to be very volatile, with more new and broken ties than stable ties every 2 weeks. We recognize that part of these changes may be due to random error or ‘noise’ in the respondents’ answers to the question about their peer network. The number of nominations was limited to five, and it may be the case that some respondents have larger pools of friends at school from which they pick five. For those students, the friends they nominate might differ between waves even if previously-mentioned ties were not broken, thus producing an over-estimation of change.

To evaluate this, we conducted a small robustness check in which we focused on the amount of change in friends that were nominated first in each wave. These friends can be expected to be one’s ‘best friend’ at the moment. Interestingly, this specific nomination also demonstrated a high turnover rate between waves. On average, 38 percent of respondents changed the first nominated friend of their school grade between waves. This finding suggests that even the friends that first come to mind change between waves for more than one in three respondents. This corroborates our impression that the grade 9 network of youth in our study is highly dynamic, and on balance, we believe that the prevalence of broken and new friendships depicted in Table 2 and the visible changes in the networks over the waves in Fig. 1 are impressive, even if occasional error occurs in the nomination of friends.

The results further demonstrated that behavioral patterns are also dynamic in the short run. A substantial portion of the respondents changed their involvement in offending in the 2 week intervals, in particular with regard to minor violence and overall offending. There were also several respondents who appeared to use alcohol or marijuana in some periods but not in others. Moreover, the amount of unstructured socializing during 2 weeks fluctuated for most of the respondents. Of course, part of these changes may also be due to random error or noise in the answers of the respondents. However, the short periods of 2 weeks between intervals make it unlikely that the respondent did not remember behaviors or projected it later or earlier in time. It also seems unlikely that they differed in their willingness to self-report offenses or substance use across 2-week time windows. Therefore, once again, we believe that on balance most of the changes in behaviors we observe in the data reflect true fluctuations between waves.

Longitudinal (SIENA) network analyses revealed several peer selection effects. We found robust structural network effects—most importantly reciprocity (choosing someone who has chosen you before), transitivity (becoming friends with friends of friends), balance (choosing others who chooses friends), and indegree popularity (choosing someone who is popular). In other words, the respondents in our particular sample of ninth grade students from a Kentucky high school reflect the general sociological laws of network dynamics very well. Respondents also displayed a strong preference for same-sex friends, but not necessarily for friends of the same race or SES. Interestingly, delinquent values appeared to play a significant role in the short-term dynamics of the investigated peer network. Specifically, respondents had a preference to make ties to others with similar values and to others with relatively delinquent values. This finding is relatively new, because only a few studies have investigated this possibility. A previous network study employing a one-year interval between waves did not find a selection effect for attitudinal selection (Weerman 2011), but a more recent study on peer beliefs about alcohol did also find selection effects for similarity in moral and cognitive beliefs about alcohol use, as well as an alter and ego effect (Ragan 2014). It is also in line with social network studies outside criminology that frequently report peer selection effects based on attitudes other than delinquent values (see for an overview Veenstra and Dijkstra 2011).

Perhaps surprisingly, actual delinquent behavior of peers did not result in a significant selection effect, contrary to the classic notion that ‘birds of a feather flock together’. We also did not find a significant selection effect for peer levels of previous offending, which corroborates the findings about current levels of peer delinquency.^23^ These findings also contradict many traditional longitudinal studies on peers and delinquency (e.g., Matsueda and Anderson 1998) and most longitudinal network studies (e.g., Haynie et al. 2014; Snijders and Baerveldt 2003; but see Weerman 2011). Perhaps the general attitude towards offending is a more visible and communicated source of information for peer selection purposes. It is possible that actual offending behavior may be too rare or erratic to become known to fellow students, while attitudes of fellow students become known by informal conversations and reactions to behavior, or stories or images on social media. Perhaps adolescents do not talk specifically about their moral beliefs, but show it in gesture, daring behavior, and deviant talk (see e.g., Granic and Dishion 2003). The relative popularity of peers with delinquent values may also be the result of this visibility in talk and hints that suggest involvement in “tough” or daring behaviors.

Also contrary to expectations, we did not find significant peer socialization effects in our study. Despite suggestions in previous literature that peer influence effects can take place over relatively short periods of time, the respondents in this study did not appear to adapt their behavior to previous offending behavior of their nominated friends in the network. We repeated this analysis with different modes in the SIENA software, and with a more restricted time period, but the absence of a significant effect was robust. These results mean that offending of friends in the school network did not influence one’s own behavior during the short research period of this study. This departs from most traditional longitudinal studies on the relation between peers and delinquency (e.g. Matsueda and Anderson 1998) and from several longitudinal network studies (e.g. Burk et al. 2007; Weerman 2011; Haynie et al. 2014) that employed relatively long term intervals between measurements. On the one hand, this finding may mean that the socialization (or balancing) processes do not emerge or do not emerge clearly enough in the short periods of our study. On the other hand, it is possible that acts of delinquent behavior by peers are simply not observed or noticed well enough to produce a short-term influence effect.

The results are more supportive of our expectation that the short-term change in behavior observed here can be influenced by situational peer mechanisms. We found that changes in delinquent behavior over the research period were related to changes in unstructured socializing, alcohol use and marijuana use.^24^ This also held for the restricted time period of wave 2 to 5, though the specific situational factors that were most important differed across the various categories of offending.

Our findings have implications for future research and theory. First of all, this study demonstrates the importance of collecting data on the short-term dynamics of peers and delinquent behavior. Delinquent behavior appears to be more erratic than traditional research suggests, and may have a short-term ‘zigzag’ pattern^25^ in which involvement in offending may go back and forth during periods of weeks. These data suggest that adolescents are involved in delinquent behavior during some of these periods, but abstain from it in others. This study also revealed that peer relations can be volatile resulting in very dynamic friendship networks, even within time frames of weeks. However, most research on the causes of delinquency employs yearly or, at best, several-months intervals. Although this traditional approach has resulted in many useful insights, it can lead to mis-attributions because it links data that may refer to different periods in time. With one-year intervals, the delinquency measure refers to a total sum of (sporadic) involvement during different periods, while the measurement of peer networks and time use with peers is a snapshot of the situation at the moment of data collection, which may well be different from the periods in which respondents actually offended. In other words, our findings imply that studies employing longer time gaps between measurements may miss the dynamic nature of this life period and may fail to capture short-term relationships between peers and behavior.

Our study resembles a study conducted by Horney et al. (1995) who related monthly fluctuations in offending to monthly fluctuations in ‘local life circumstances’ like going to work or school, living together, and heavy use of alcohol or drugs. In our study, changes in friendship networks and in time use patterns and substance use represent an adolescent version of ‘changes in local life circumstances’ that have been studied by Horney et al. among adults. More generally, the peer and time use measures that have been the focus of this paper may be part of a wider set of variables that have important implications for short term fluctuations in offending—for adolescents as well as adults. It would be interesting to expand the study of short term changes in delinquency to a wider set of variables and people in future research.

Second, our study has implications for theorizing about peers and delinquency. If peer networks and behavior are so dynamic, then how exactly are they influencing each other? To unravel this, it may be useful to distinguish between longer-term and short-term peer processes (see also Hoeben and Weerman 2016). As mentioned earlier, longer-term peer processes include social learning mechanisms through reinforcements and imitation (Akers 2009), or peer socialization through the transmission of norms and deviant definitions (Sutherland 1947). Peer selection is also a process that takes some time, but apparently does not have to take months or years to unfold. Peer processes in the very short run are situational mechanisms like status threats, fear of ridicule and ‘rowdy behavior’ (Warr 2002; Short and Strodtbeck 1965) or opportunities and situational inducements during unstructured socializing (Osgood et al. 1996).

Although the use of shorter measurement intervals than usual provides an advancement over traditional methods with one year intervals, we cannot be certain that the time frame of this study, 10 weeks in total with 2-week intervals, is equally well suited to capture selection, socialization and situational effects. For example, we did not find a significant socialization effect of peer delinquency on one’s own offending, but it might be the case that such an effect takes more time to materialize than the period we investigated. It is also possible that general changes in time use or lifestyles take hold over a relatively longer period of time, while truly situational effects operate at time intervals even shorter than 2 weeks. Future studies may include long term as well as short term measurements of peer delinquency to explore which time frames are relevant for different peer mechanisms (see Horney, 2001). Such studies may also include very short time measurements of situational circumstances, like the hourly time intervals that are employed in space–time-budget interviews (see Hoeben et al. 2014; Wikström et al. 2012). In general, it is essential that the longitudinal data structure is linked to the processes that are believed to generate the relationships of interest (Collins 2006).

More generally, we concur with the recommendation of Agnew (2011) that criminologists should devote more attention to the causes of crime during different time frames. He suggested to distinguish at least three temporal levels: long-term averages, short-term deviations around these averages (hours and days), and situational deviations (seconds to minutes). Whether these are the most relevant units for the relationship between peers and delinquency, or whether relevant changes may also occur within periods of weeks and months needs to be elaborated in future theorizing and scrutinized in empirical research.

Despite these implications, this study has some important limitations. First of all, the sample size is small: one school grade in one particular secondary school in a rural area. This means that definitive conclusions about the presence and strength of effects are not possible. We may have missed effects that are actually present in reality because of our small sample size, which limits statistical power, and some of the effects we find may be coincidental. However, it is remarkable that we did find very robust structural network effects, even within our limited study, that were consistent with previous network studies, and we also found several robust selection effects and an expected pattern of situational effects. Such findings suggest that conclusions drawn from our results have some validity with respect to replication of previous research.

A second limitation is that our measurement of unstructured socializing is less than optimal. The current study employs the method of stylized questioning to measure unstructured socializing (see Hoeben et al. 2014), asking respondents in retrospect about their time use. This method may result in problems with recall, although we believe that this will be relatively minor in the current study because our measurement of unstructured socializing refers to a period of only 2 weeks. Future research might employ more precise measurements like time diary methods, such as the Space Time Budget interview (see Wikström et al. 2012; Hoeben and Weerman, 2016), or experience sampling to systematically measure in detail what adolescents are doing at randomly selected time points during a given day (see Csikszentmihalyi et al. 1977).

A third limitation is that our measurement of the peer network was limited to ninth-grade fellow school students. In reality, adolescents have friends not only from their own school grade, but also from other grades, and more importantly from outside their school. It is probable that these friends will also exert influences on offending and that the wider peer network is as dynamic as the network in the school. We limited our study to the within-grade network because this is the most feasible way to follow a complete network over time, while including each of its participants. Although it is not easy to accomplish, future research might expand knowledge on peer networks by gathering data about complete school or neighborhood networks.

Finally, a fourth limitation is that we may have left out variables that are relevant in understanding short-term changes in peer networks and behavior. For example, past research suggested that classroom composition can be very relevant for peer selection, simply because adolescents who spend a lot of time in each other’s vicinity have a higher chance of becoming friends (e.g., Weerman 2011). The respondents of our sample did not have distinct classes with their own classrooms, but changed classrooms for each subject; nevertheless they may have been more in contact with some fellow ninth graders than with others.

In closing, the current study is not meant to provide definitive conclusions about selection, socialization and situational peer effects with regard to delinquency, and we are also not able to provide the final judgement about the mixed findings from previous SIENA studies (in fact, our findings add some new dimensions to the puzzle). Such goals are impossible given the limitations regarding sample size, design and measurements. Instead, we aimed to explore whether using and analyzing shorter time intervals than usual in criminological research can offer new and different insights on the dynamics of peers and delinquency. Our findings demonstrate that this is indeed the case. Unlike network studies using longer time intervals between waves, we found neither selection nor socialization effects for peer delinquency, but instead found other peer selection effects and influences to be important on the short run: structural network dynamics and selection effects of peer delinquent values, and situational effects from unstructured socializing with peers and substance use. Our study also demonstrated that peer networks and behavior are more volatile by nature than longer-term research seem to recognize. In short, we believe that future research with short time intervals is warranted to further unravel the highly dynamic relation between peers, time use, and delinquent behavior in adolescence.

## Appendix 1

See Table 5.

**Table 5:** SIENA estimation of network and behavior dynamics of total offending variety: parameter estimates and standard errors wave 2–5 (155 persons; 755 observations)

Parameter	Estimate	SE
Network structural effects		
Outdegree/density effect	−3.416**	(0.144)
Reciprocity	1.977**	(0.123)
Transitive ties	0.915**	(0.092)
Balance	0.121**	(0.021)
3-cycles effect	−0.177	(0.119)
Indegree popularity	0.058**	(0.018)
Other preferences/selection effects		
Alter female	0.049	(0.089)
Ego female	0.146	(0.102)
Same sex	0.632**	(0.083)
Alter minority	0.086	(0.126)
Ego minority	−0.082	(0.127)
Same race	0.199^+^	(0.116)
SES alter	0.049	(0.030)
SES ego	−0.007	(0.036)
Similarity in SES	0.317	(0.239)
School attachment alter	−0.036	(0.070)
School attachment ego	−0.050	(0.080)
Similarity school attachment	0.155	(0.223)
Delinquent values alter	0.174*	(0.082)
Delinquent values ego	−0.026	(0.088)
Similarity in delinquent values	0.533**	(0.252)
Low self-control alter	−0.082	(0.071)
Low self-control ego	−0.207**	(0.080)
Similarity in low self-control	−0.113	(0.229)
Number of friends in higher grades alter	0.059	(0.045)
Number of friends in higher grades ego	−0.024	(0.052)
Similarity in # of friends in higher grades	0.476^+^	(0.249)
Previous offending alter	0.093	(0.096)
Previous offending ego	0.250*	(0.102)
Similarity in previous offending	0.637	(0.608)
Behavioral dynamic selection effect		
Offending alter	0.040	(0.112)
Offending ego	0.059	(0.125)
Similarity in offending	−0.148	(0.379)
Effects on offending: basic, controls		
Linear shape (trend)	−1.510**	(0.358)
Quadratic shape	0.029	(0.256)
Sex	0.017	(0.343)
Race	0.601	(0.386)
SES	0.011	(0.128)
School attachment	−0.196	(0.301)
Delinquent values	−0.012	(0.257)
Low self-control	0.186	(0.269)
Number of friends in higher grades	−0.332	(0.216)
Previous offending	−0.049	(0.242)
Effects on offending: dynamic socialization effect		
Average similarity (socialization effect)	−0.868	(2.228)
Effects on offending: situational effects		
Unstructured socializing during period	0.053^+^	(0.029)
Drinking alcohol during period	1.367**	(0.526)
Using marijuana during period	0.989	(0.728)

^+^ *p* < .10; * *p* < .05; ** *p* < .01

## Appendix 2

See Table 6.

**Table 6:** SIENA estimation of network and behavior dynamics for three types of offending: parameter estimates and standard errors (n persons = 150; n observations = 550)

Parameter	Minor violence		Serious violence		Vandalism and property offending	
	Estimate	SE	Estimate	SE	Estimate	SE
Behavioral selection effect						
Similarity in offending	−0.185	(0.160)	−0.187	(0.323)	0.165	(0.348)
Effects on offending: basic, controls						
Linear shape (trend)	−1.127**	(0.356)	−2.789*	(1.354)	−3.761*	(1.714)
Delinquent values						
Effects on offending: socialization and situational effects						
Average similarity (socialization)	0.293	(0.736)	0.839	(1.837)	0.085	(1.994)
Unstructured socializing period (sit.)	0.132*	(0.054)	0.112^+^	(0.063)	0.076	(0.081)
Drinking alcohol during period (sit.)	1.215	(0.894)	1.599	(1.084)	2.595**	(0.923)
Using marijuana during period (sit.)	2.579	(2.625)	1.707	(1.164)	2.226*	(0.988)

^+^ *p* < .10; * *p* < .05; ** *p* < .01

## Footnotes

1 With the term ‘mechanism’, we refer to an underlying internal or external process that explains why peer variables are related to offending behavior. We do not mean that condition or characteristic X directly leads to Y, but rather that X increases the chance of Y. We also do not aim to identify causal chains with mediating variables. Instead, with ‘mechanism’ we aim to identify the ways in which peers, situations and behavior are expected to influence each other.

2 The baseline sample included 541 participants, approximately 78% of the school’s student population.

3 Active parental consent was obtained through face-to-face contact between study investigators and the parents during a 1-week registration fair at the school in July, 2006. The school district requires that parents accompany their child to this fair each summer in order for them to register for classes. Only two parents who were contacted at this fair refused to let their child participate in the study. Most of those for whom consent was not obtained were non-attendees or were “missed” by investigators at the fair. In other words, “no contact” as opposed to “refusal” was the modal reason for non-participation.

4 For instance, U.S. Census estimates at the time of survey administration suggested that the county population was 90.9% white and 7.5% African American, compared with 90.1 and 7.3% for the state of Kentucky overall. Other census figures revealed compatibility between residents within the county of the sampled school and Kentucky residents overall in terms of educational attainment and socio-economic level: 73.3% of Northern County (pseudonym) residents were high-school graduates compared to 74.1% for the state; and the median household income in Northern County was $30,195 versus $33,672 in Kentucky (U.S. Census). School-district data also reveal similarity between Northern County and the overall Commonwealth. For example, the race distribution in the school during 2006 was: 90.1% white, 9.5% African-American and 0.5% other race compared to 86.2% white, 10.4% African-American and 3.4% other race in schools statewide.

5 The average number of nominated close friends varied slightly across waves: 4.07 in the first wave, 3.60 in the second, 4.01 in the third, 4.17 in the fourth, 4.03 in the fifth wave.

6 This was quite stable across waves; averages were 2.95 in the first wave, 2.75 in the second, 2.83 in the third, 2.87 in the fourth, 2.81 in the fifth wave.

7 At wave 1, which was administered during the first week of school, the reference category was “during the past summer break” as opposed to “during the past 2 weeks.” Response categories for the baseline measurement of offending ranged from 1 = 0 days to 7 = 60 or more days.

8 We explored whether we could run SIENA analyses with delinquency scales based on summation of the items, but this resulted in major convergence problems.

9 The categorization was based on a factor analysis on dichotomized items using promax rotation. The three factor solution was chosen because the scree plot showed a bend at three after which the eigenvalue and explained variance did not change much anymore.

10 Dichotomizing was preferable in this analysis since using the original response scale was not possible in SIENA.

11 As with offending, the time reference for the wave 1 survey was “during this past summer break” as opposed to “during the past 2 weeks.”.

12 On average, 55% of the respondents had at least one or more friends from higher grades.

13 The SIENA homepage (http://stat.gamma.rug.nl/snijders/siena.html) offers additional information and links to program downloads.

14 An exception occurred regarding the combination of transitive triplets and transitive ties. We chose to include the transitive ties effect instead of the more popular ‘transitive triplets’ effect, because it is a more generalized measure of triadic closure and because its estimated effect appeared to be more substantive.

15 A positive parameter means that behavior that is not at the mean intensifies further away from the mean over time, while a negative parameter represents that there is a tendency of behavior to be drawn towards the mean over time.

16 This is the most common indicator for peer influence used in RSiena in which the potential influence of alters is the same regardless of the number of alters. There are two alternative possibilities to model peer influence, the ‘total similarity’ effect (the extent to which respondents become similar to the total of their nominated alters’ offending levels) and the ‘average alter’ effect (a simplified linear effect of the average offending level of nominated alters).

17 These values are assumed to be constant between waves, while the networks and behaviors are assumed to change continuously over each micro-step.

18 These impressions are corroborated by the Jaccard Indices, which are often used as a measure of stability in network data. This index is 0.43 in the period between wave 1 and 2; 0.48 between wave 2 and 3; 0.51 between wave 3 and 4; and 0.52 between wave 4 and 5. A Jaccard index of around .50 indicates quite a bit of change (but not too much to use SIENA modeling). The increasing value across the waves indicates greater stability over time.

19 This is somewhat contrary to our initial expectation that having older friends might lead to increased delinquent opportunities and group influences. One explanation is that an eventual negative influence from older peers already occurred before the study period of 10 weeks, during summer break. In that case, their involvement in offending already peaked during the first wave and declined afterwards. That explanation is partly supported by the findings from the robustness check with analyses restricted to the sample from wave 2 to wave 5. In that SIENA analysis, the effect of friends from higher grades is not significant anymore, though still negative. Another explanation is that older friends have protective effects on delinquent behavior because they are less involved in offending than the ninth graders that were studied. This explanation is supported by the finding from the larger Rural Substance Abuse and Violence Project that the overall growth parameter in delinquency for this sample was negative (see Ousey and Wilcox 2007).

20 We reran the analyses with two alternative indicators for peer socialization (the ‘total similarity’ effect and the ‘average alter’ effect. These alternative models did not lead to different results than those we present.

21 The estimated effect is 0.048 per unit on the unstructured socializing scale, the average standard deviation for that scale is 4.8.

22 These specific results are available from the first author upon request.

23 We only found a marginally significant ‘ego-effect’ of previous delinquency, indicating that respondents who were more delinquent in the previous year were more active in making friendships.

24 The effects of marijuana use may point towards a direct psycho-pharmaceutical effect of drug on behavior, but the research literature on this is inconclusive and methodologically superior studies tend towards an absence or even a disinhibiting effect of cannabis (Myerscough and Taylor 1985). Alternatively, it may be possible that the non-normative use of cannabis in this relatively young sample is co-occurring with forms of delinquency during periods in which adolescents are oriented towards breaking the rules or towards acting ‘rowdy’ (see Warr 2002; Csikszentmihalyi and Larson 1984).

25 The term comes from Laub and Sampson (2003) who described long-term zigzag patterns in offending during the complete life course.
